# A waterproof, low-cost dressing system reduces postoperative wound dressing changes in primary total hip arthroplasty: An efficacy study

**DOI:** 10.3389/fsurg.2022.966874

**Published:** 2022-08-11

**Authors:** Shilong Su, Chenggong Wang, Fawei Gao, Yihe Hu, Da Zhong, Pengfei Lei

**Affiliations:** ^1^ Department of Orthopedics, Xiangya Hospital, Central South University, Changsha, China; ^2^ Department of Orthopedics, Peking University Third Hospital, Haidian, Beijing, China; ^3^ Department of Orthopedics, The First Affiliated Hospital, College of Medicine, Zhejiang University, Hangzhou, China; ^4^ Hunan Engineering Research Center of Biomedical Metal and Ceramic Implants, Xiangya Hospital, Central South University, Changsha, China; ^5^ Hunan key laboratary of aging biology, Xiangya Hospital, Central South University, Changsha, China

**Keywords:** dressing, dressing changes, hip arthroplasty, waterproof, efficacy

## Abstract

**Backgrounds:**

Postoperative wound complication is a major risk factor for the development of Periprosthetic joint infection. We innovatively invented a new dressing system to reduce the occurrence of postoperative wound complications and improve the quality of life of patients after total hip arthroplasty.

**Methods:**

A total of 120 patients who underwent primary unilateral total hip arthroplasty were enrolled in this study. The data collected included the number of dressing changes, costs of the dressings, postoperative hospital stay, The Visual Analogue Scale (VAS) score, The Harris Hip Score (HHS), ASEPSIS score, The Stony Brook Scar Evaluation Scale (SBSES), wound complications, the frequency of showers and satisfaction. Data were statistically analyzed.

**Results:**

The average number of dressing changes was 0.74 ± 0.46, while the average postoperative hospital stay was 3.67 ± 0.97 days. The average cost of the new dressings throughout a treatment cycle was 57.42 ± 15.18 dollars. The VAS score decreased from 5.63 ± 1.09 before the operation to 0.88 ± 0.54 one month after the operation. The HHS score increased from 70.18 ± 7.84 before the operation to 80.36 ± 4.08 one month after the operation. The results of the four indexes of the ASEPSIS score were all 0. The SBSES score was 3.55 ± 0.61 at two weeks after the operation, and 4.38 ± 0.71 at one month after the operation. No wound complications were recorded until one month after the operation when the satisfaction rate was 92.53 ± 3.62%.

**Conclusion:**

In this study, we have invented a new dressing system for surgical wounds after total hip arthroplasty and confirmed its efficacy.

**Chinese Clinical Trial Registry:**

ChiCTR2000033822, Registered 13/ June/2020

## Introduction

Total hip arthroplasty is one of the most common orthopedic procedures, and as the population ages, the incidence increases every year. Studies report that among patients undergoing total hip arthroplasty, the incidence of periprosthetic joint infection (PJI) ranges between 0.59% and 2% (1, 2). PJI is the most serious complication of joint replacement (3), which causes physical, emotional, and economic losses to patients, hospitals, and the health care system (4). Numerous risk factors for PJI are reported in the literature, including superficial wound complications (5, 6), indicating that proper wound care is essential for the prevention of PJI. Currently, the traditional dressing using aseptic gauze and plastic tape is used after orthopedic surgery. In some cases, wound complications such as erythema and blisters have been observed, resulting in an increased risk of wound pain and infection. Therefore, the traditional gauze dressing is not an ideal dressing for hip arthroplasty.

Calcium alginate dressing, as a new type of wound dressing, can effectively control exudation, thus prolonging the dressing change time (7). Besides, it forms a gel and keeps the wound moist, and can also release calcium ions to promote hemostasis and inhibit bacterial growth (8). Studies have shown that moist wounds heal faster and have less pain (9). Thus, calcium alginate dressing has good application prospects. Currently, calcium alginate dressings are often used in combination with gauze dressings. However, this cannot overcome the shortcomings of gauze dressings, and also limits the advantages of calcium alginate dressings, such as prolonging the time of dressing change.

To solve this clinical problem, we innovatively used IV3000 film and calcium alginate dressing in surgical incision management of patients undergoing hip arthroplasty. IV3000 film is a kind of dressing film for intravenous catheterization, with high moisture permeability (10), good waterproof performance, inhibits bacterial colonization (11), has good skin adhesion, no friction with the skin, and almost no pain at removal (12). The combined use of the two not only makes use of the advantages of a calcium alginate dressing, but also makes use of the characteristics of IV3000 film.

This clinical trial was designed to confirm the clinical efficacy of the new dressing system. The trial was evaluated by recording the number of postoperative dressing changes, postoperative hospitalization days and medical costs, wound complications and healing, functional recovery and quality of life of patients, the frequency of showers and self-evaluation of patients' satisfaction.

## Patients and methods

The study was conducted according to the Declaration of Helsinki principles (as revised in 2013) and approved by The Medical Ethics Committee of Xiangya Hospital of Central South University and written informed consent was obtained from all the patients. The study was registered at www.chictr.org.cn (ChiCTR2000033822, Registered 13/ June/2020).

The inclusion criteria of patients were as follows: 1. Aged 18 to 85 years old, 2. osteoarthritis and osteonecrosis of the femoral head were diagnosed by physical examination and imaging data, 3. The patient was to undergo primary unilateral total hip arthroplasty. Patients who previously had joint surgery on any hip joint, have obvious scars on any hip joint, suffer from skin diseases such as psoriasis, eczema, or dermatitis, and those who cannot complete regular follow-up were excluded from the study.

Between June 20, 2020, and November 20, 2020, a total of 120 patients were enrolled in the study. There were 59 males and 61 females, with a median age of 57.17 ± 12.86 years old (range 21–75 years old). The demographic characteristics were shown in Table 1. All the operations were performed by an experienced joint surgeon. The operation was performed using a standard posterolateral approach and the prostheses were all biological.

**Table 1:** Demographic characteristics of the patients.

Characteristics	Subjects (*n* = 120)
Age, year	
Median age	57.17 ± 12.86
Range	21–75
Age distribution, year, No (%)	
18–50	12 (10%)
50–70	102 (85%)
70–85	6 (5%)
Gender	
Males	59 (49.17%)
Females	61 (50.83%)

### Application of the new dressing system

**Figure 1:**
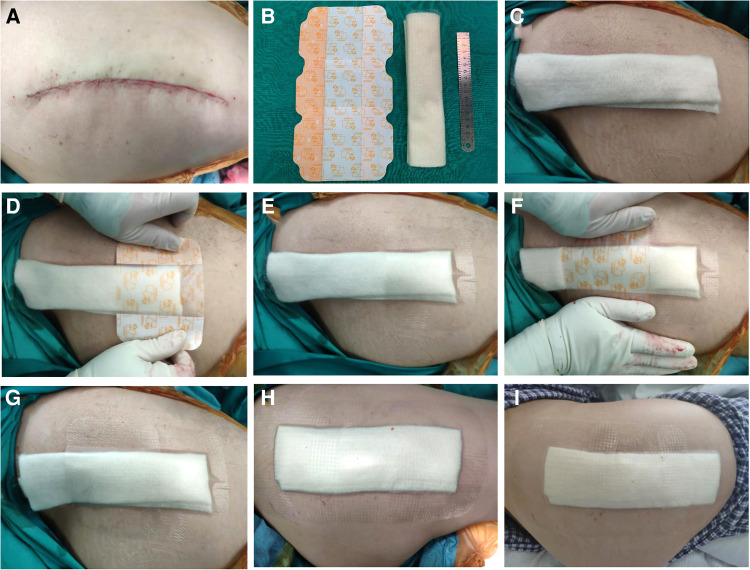
(**A**) The wound was sutured and deiodized. (**B**) Folded calcium alginate dressing and three IV3000 films. (**C**) Cut the calcium alginate dressing to both ends slightly longer than the incision 1 cm. (**D**–**H**) According to the length of the incision, three IV3000 films were selected and applied in the order from the distal end to the proximal end of the limb. The two ends of the film were slightly longer than the incision about 4 cm, and the latter film was overlapped and the previous one was about 1 cm. There are no air bubbles between the films, and the skin and stick closely to the skin. (**I**) After the patient took a bath according to his own habits, the dressing was not affected.

Prophylactic antibiotic cefoxitin was routinely used 30 min before the operation. The standard three-layer continuous suture method was used in all patients during the operation. The articular capsule was sutured continuously with 2# absorbable knot-free unidirectional barb suture (Quill, Surgical Specialties Corporation, New York, USA), subcutaneous tissue was sutured with 0# absorbable knot-free bi-directional barb suture (Quill, Surgical Specialties Corporation, New York, USA), and intradermal was sutured with 3-0 absorbable knot-free bi-directional barb suture (Quill, Surgical Specialties Corporation, New York, USA). The usage of the new dressing system: 1. After the surgical incision was sutured, 10 cm of skin around the incision was thoroughly de-iodinated with 75% alcohol (Figure 1A). 2. The calcium alginate dressing (Algisite M, Smith & Nephew, London, UK) was folded into three layers in the direction of the long axis and properly cut to a length range slightly longer than the surgical incision of 1 cm at both ends (Figures 1B,C). 3. based on the length of the incision, three to four IV3000 films (Smith&Nephew, London, UK) were selected and applied in the direction from the distal end to the proximal end of the limb (Figure 1D). The two ends of the film were slightly longer than the incision by about 4 cm, and the latter film was overlapped and the previous one was about one cm (Figures 1E–G). There were no air bubbles between the films and the skin, and they stuck closely to the skin (Figure 1H). After the operation, all patients adopted the same nursing measures: routine application of prophylactic antibiotic cefoxitin for three days, and subcutaneous injection of enoxaparin sodium 4000IU to prevent deep venous thrombosis. Patients with the new dressing system did not need to change their dressings if there was no obvious large amount of exudation, no scratches, or crimps, and two weeks after the operation, the dressing would be removed. Patients were able to shower normally after the operation (Figure 1I).

### Data collection

Data were collected in four parts: the number of dressing changes and cost of dressings, pain and function scores, wound scores and complications, and shower frequency and satisfaction.

### The number of dressing changes and cost of dressings

Patients were discharged only when they met stringent standards, including the ability to perform independent personal care, walk at least 70 meters on crutches, get in and out of bed and get up from chairs, and were managed with oral pain relief (13). The postoperative hospital stay was also recorded and calculated as the whole day, and the part less than one day was considered as one day. After discharge, the patients were assigned to a chat group to take photos and upload and evaluate the dressing under the guidance of the medical staff. Two weeks after the operation, all patients were not covered with a dressing, the wound was wiped with 75% alcohol three times a day for three days, and the total number of dressing changes were recorded. Besides, the medical expenses incurred by patients using the new dressings were recorded to understand the average cost of the new dressings throughout the treatment cycle.

### Pain and function score

The Visual Analogue Scale (VAS) score, The Harris Hip Score (HHS) were used to record the pain and function of patients, and to evaluate the perioperative changes. VAS score (14) is a one-dimensional measurement of pain intensity, which is widely used in different adult populations. The VAS score was used to record pain and is a horizontal line of fixed length, 100 mm. The end is defined as the limit of pain to be measured, from left (0) to right (10). HHS (15) was developed to evaluate the results of hip surgery and to evaluate various hip disabilities and treatments in the adult population. HHS assesses pain, function, deformity, and range of activity and each project has a unique digital scale. The highest score for HHS is 100. The higher the HHS, the less the dysfunction. The time point of the evaluation was recorded within one week before the operation, and one month after the operation.

### Wound score and complications

ASEPSIS score is a commonly used wound assessment score (16), which consists of an objective wound assessment section, a section on wound treatment, and a section on the consequences of infection. The objective wound assessment part of the ASEPSIS score (17) was used in the current study because the intentions were to only evaluate the clinical appearance of the wound. SBSES score, proposed by Singer et al in 2007 (18), is a wound evaluation scale used to measure the cosmetic effect of a wound, including the width, height, color, suture marks, and overall view of the scar. The score of each index is 0 or 1, and the total score is calculated, ranging from 0 (worst) to 5 (best). The ASEPSIS score and the SBSES score were recorded at seven days and one month after the operation. Follow-up was based on the photos taken or on-site observation records. At the same time, the wound complications of the patients in each period were recorded and photographed within one month after the operation.

### Shower frequency and satisfaction

A questionnaire was developed to conduct the shower frequency and satisfaction survey (Table 2). One month after the operation, the patients filled the questionnaire based on their actual situation. The questionnaire recorded patients' satisfaction based on eight parameters, including their comfort with dressings, ability to take a shower, pain treatment, doctor visits, hospital stay, number of dressing changes,hospitalization costs, and satisfaction with the overall experience. The parameters were all measured in numerical terms, with a score of 0 to 10, and a maximum score of 80.

**Table 2:** Questionnaire record table.

	1	2	3	4	5	6	7	8	9	10
Comfort with dressings
Ability to take a shower
Pain treatment
Doctor visits
Hospital stay
Number of dressing changes
Hospitalization costs
Overall experience
Shower frequency last month										

Data were collected by one of the researchers who was not directly involved in either the experimental design or surgery. All quantitative data were expressed as mean ± standard deviation. A paired t-test was used to compare the two groups. *P* < 0.05, was considered to be statistically significant. SPSS25.0 software (SPSS, USA) was used to perform statistical analysis.

## Results

### The number of dressing changes and cost of dressings

The average number of dressing changes was 0.74 ± 0.46, and the average postoperative hospital stay was 3.67 ± 0.97 days. The application of the new dressing system required an average of one calcium alginate dressing and three IV3000 films, and the calculated cost of one dressing change was 33 dollars. The average cost of the new dressings throughout a treatment cycle was 57.42 ± 15.18 dollars.

### Pain and function score

VAS, and HHS were used to record the pain, and function of the patients, and the evaluation time was set within seven days before the operation, and one month after the operation. The VAS score decreased from 5.63 ± 1.09 before the operation to 0.88 ± 0.54 one month after the operation. The HHS score increased from 70.18 ± 7.84 before the operation to 80.36 ± 4.08 one month after the operation (Table 3).

**Table 3:** The score results of VAS, HHS.

Variable	Preoperative	One month postoperatively	*P* value
VAS	5.63 ± 1.09	0.88 ± 0.54	<0.001
HHS	70.18 ± 7.84	80.36 ± 4.08	<0.001

VAS, Visual Analogue Scale; HHS, The Harris Hip Score.

### Wound score and complications

**Figure 2:**
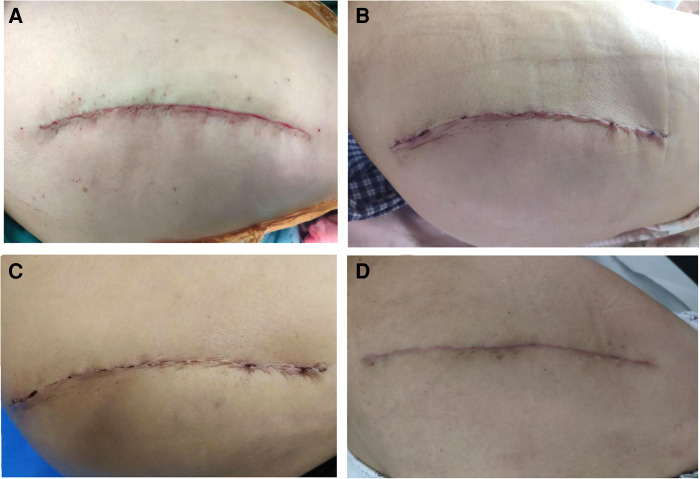
(**A**) Shows wound sutured during the operation. (**B**) There was no obvious ecchymosis, swelling, and exudation in the wound three days after the operation. (**C**) The wound healed completely two weeks after the operation. (**D**) One month after the operation, the wound of the patient showed that the scar was smooth, consistent with the color of the surrounding skin, and the overall appearance was satisfactory.

During the use of the new dressing system, normal bathing did not affect the dressing, and the waterproof performance was good. The results of serous discharge, erythema, purulent discharge, and wound defect defined by the ASEPSIS score were all 0 (Table 4). The SBSES score was 3.55 ± 0.61 at two weeks after the operation and 4.38 ± 0.71 at one month after the operation (Table 4). The wound appearance gradually improved with the prolongation of recovery time. No wound complications were recorded until one month after the operation. The wounds healed well and the patients described their scars as comfortable and satisfactory in appearance (Figure 2).

**Table 4:** The score results of the ASEPSIS and SBSES.

	Two weeks postoperatively	One month postoperatively	*P* value
ASEPSIS			
Serous discharge	0	0	1.000
Erythema	0	0	1.000
Purulent discharge	0	0	1.000
Wound defect	0	0	1.000
SBSES			
Width	0.69 ± 0.46	0.81 ± 0.40	0.057
Height	0.88 ± 0.33	0.90 ± 0.30	0.640
Color	0.06 ± 0.24	0.69 ± 0.46	<0.001
Suture marks	1.00 ± 0.00	1.00 ± 0.00	1.000
The overall view	0.95 ± 0.22	1.00 ± 0.00	0.025
Total score	3.55 ± 0.61	4.38 ± 0.71	<0.001

SBSES, Stony Brook Scar Evaluation Scale.

### Shower frequency and satisfaction

According to the questionnaire results, the patient's shower frequency was shown in Table 5, 10 patients (8.33%) did not take showers because they were afraid of getting wound infections. Most patients (95/120, 79.17%) took showers once per day. And the satisfaction score was 73.86 ± 2.81, the full score was 80, and the satisfaction rate was 92.53 ± 3.62%.

**Table 5:** The results of the shower frequency.

Frequency	Number	*n*%
No shower	10	8.33%
Twice per day	5	4.17%
Once per day	95	79.17%
Every 2 days	7	5.83%
Every 3 days	2	1.67%
Every 4 days	1	0.83%

## Discussion

PJI is a serious complication of joint replacement surgery and causes serious medical and economic burden to patients and society. Previous studies (5, 6, 9, 19) show that complications of the surgical wound are a major risk factor for PJI, thus, the management of surgical wounds is very important. Considering the particularity of the wound after hip arthroplasty, a combination of the gauze and adhesive tape, which is widely used in our hospital is not appropriate. First, the absorption effect of the exudate by the gauze dressing is not good, and it is easy to soak, and this increases the frequency of dressing change. These are all risk factors for wound infection. Second, gauze dressings often adhere to the wound after wetting, causing skin damage and pain during wound dressing change. Third, the surface of the gauze dressing is rough and inelastic, and multi-layer coverage can cause bloated wounds. During postoperative hip movement rehabilitation exercise, this may cause obstacles and constant friction which also causes blisters. Fourth, gauze dressings are usually not waterproof, therefore, patients are likely to experience difficulties in having a normal bath and skin cleaning after the operation.

To overcome the shortcomings of using gauze dressings, numerous new dressings have emerged. As new dressings for primary joint replacement wounds, incision negative pressure wound therapy (iNPWT) (20, 21) and silver-impregnated occlusive dressings have been widely recognized for their advantages in reducing the incidence of wound complications and peri-prosthetic infection. The literature also reported their advantages over traditional wound dressings in terms of times of dressing change, postoperative hospital stay and cost-effectiveness. In terms of the number of dressing changes, the average number of dressing changes in the iNPWT group was 2.5 (20) and that in the silver-impregnated occlusive dressings group was 1.3 (22); However, the average number of dressing changes in our new dressing system was 0.74, which significantly reduced the number of dressing changes. Some scholars (23) have reported that if the dressing is not often disturbed, the risk of infection is reduced, and the wound dressing maintains the wound near the core body temperature, which helps the healing process. The new dressing system is simple and portable, does not cause pain when changing the dressings, has a beautiful appearance, and has elastic changes with flexion and extension during postoperative exercise, which does not hinder rehabilitation activities. The studies (20, 22) reported that the average postoperative hospital stay was 3.8 days for the iNPWT, 6.3 days for the silver-impregnated occlusive dressings and 3.7 days for our new dressing system. These new dressings significantly shorten the postoperative hospital stay, which is also in line with the requirements of the concept of enhanced recovery after surgery (ERAS): short hospitalization days, quick postoperative recovery.

As for the cost of dressings that we are more concerned about, the high cost of some new dressings is a major obstacle to their wide application. In the iNPWT group, the average dressing cost in the 7-day treatment cycle was 125 pounds. The silver-impregnated occlusive dressings can cost up to 38.05 dollars for a single change (24). The average cost of our new dressing system throughout a treatment cycle was 57.42 ± 15.18 dollars, which is lower compared with that of other new dressings and is comparable to the traditional gauze dressings. More importantly, taking advantage of the waterproof and breathable properties of the IV3000 film, patients can take a shower normally after the operation, which is of great significance. This is the advantage that other dressings do not have at present. Patients are required to prepare the skin regularly and take a shower the day before the operation, which significantly reduces the risk of bacterial infection in the skin around the surgical incision. Similarly, it is also important to take a shower and wash the skin after the operation, which cannot be achieved with gauze dressing but is achieved with the new dressing system. Normal shower after the operation not only cleans the skin around the wound, reduces bacterial colonization, reduces the risk of wound infection, but also improves the quality of life of patients after the operation, and patient satisfaction.

ASEPSIS score and SBSES score were used to evaluate wound healing and possible wound complications. The results showed that there were no wound complications one month after the operation, and the wound healed well based on the objective score of the wound. The SBSES scored highly in the evaluation of the appearance of the wound, and the patients reported the satisfactory appearance of the wound scar. The satisfaction survey showed that the patients' satisfaction rate was more than 90%, indicating that the new dressing system can provide a good experience for patients.

In this study, the results confirm the clinical efficacy of the new dressing system for the wound after total hip arthroplasty. The new dressing system has various advantages, including a reduced number of dressing changes, waterproof and of low cost. However, these advantages need to be verified using a larger sample size in clinical randomized controlled trials which are underway.

## Conclusion

In this study, we have invented a new dressing system for surgical wounds after total hip arthroplasty and confirmed its efficacy.

## Data Availability

The original contributions presented in the study are included in the article/Suplementary Material, further inquiries can be directed to the corresponding author/s.
